# Herpes Zoster Ophthalmicus: Disease Spectrum in Young Adults

**DOI:** 10.4103/0974-9233.80710

**Published:** 2011

**Authors:** Noopur Gupta, Ritika Sachdev, Rajesh Sinha, Jeewan S. Titiyal, Radhika Tandon

**Affiliations:** Cornea and Refractive Surgery Services, Dr. Rajendra Prasad Centre for Ophthalmic Sciences, All India Institute of Medical Sciences, New Delhi, India

**Keywords:** Herpes Zoster Ophthalmicus, Human Immunodeficiency Virus Infection, Immune Status, Young Adults

## Abstract

**Purpose:**

To establish the clinical profile of herpes zoster ophthalmicus (HZO) in adults younger than 40 years and correlate the clinical manifestation with their immune status.

**Materials and Methods:**

A retrospective chart review was performed of patients younger than 40 years who presented with HZO. Data were collected on demographics, medical history, clinical presentation, results of serological investigations, and visual outcome.

**Results:**

The study cohort comprised 18 subjects with a mean age of 29.7 ± 6.2 years. Ophthalmic features included lid edema, ptosis, cicatricial lid deformities, sclerokeratitis, peripheral ulcerative keratitis, neuroparalytic keratitis, keratouveitis with concomitant glaucoma, secondary bacterial keratitis and superficial punctate keratitis with dry eye, optic neuritis, and trochlear nerve palsy. Eight of 18 (44.4%) subjects were found to be positive for Human Immunodeficiency Virus (HIV). Disseminated herpetic lesions were seen present in 5 (63%) of these 8 subjects. Postherpetic neuralgia was noted in 6 (75%) of 8 HIV-positive subjects and in 1 HIV-negative patient. Final visual acuity was 20/40 or better in 90% of the immunocompetent subjects and 20/200 or worse in 100% of the HIV-positive subjects.

**Conclusions:**

Immunocompetent young adults do present with features of HZO. However, the disease spectrum in HIV-negative patients is localized, less severe, and more amenable to therapy as compared with young adults with HIV.

## INTRODUCTION

Herpes zoster is characterized by reactivation of the varicella–zoster virus lying dormant in the spinal or cerebral sensory ganglia and is commonly seen in patients with depressed cellular immunity, such as the elderly, patients on immunosuppressive therapy, and patients with lymphoma or positive HIV status.^1^,^2^ The incidence and severity of herpes zoster increases with advancing age, especially after the seventh decade.^3^

With the emergence of the acquired immune deficiency syndrome (AIDS) pandemic, adults younger than 40 years are increasingly presenting with herpes zoster due to HIV.^4^ The relative risk of herpes zoster is at least 15 times greater in men with HIV than in men without HIV.^5^ The present study was undertaken to highlight the clinical profile of herpes zoster ophthalmicus (HZO) in young adults and evaluate differences between the presentation and final outcome in patients who were HIV seropositive and those who were immunocompetent.

## MATERIAL AND METHODS

A retrospective analysis of available records of young adults (<40 years old) with HZO was performed. Clinical features, results of blood laboratory workup, and treatment prescribed were noted. Repeat serological investigations were ordered, as appropriate, in patients who remained in follow-up.

## RESULTS

The clinical data of 18 young adults presenting with features of HZO were evaluated. The average age of the cohort was 30 years (range, 21–39 years). There was a predominance of males (72%) among the young adults presenting with HZO [Table 1]. A positive history of varicella in childhood (less than 10 years old) was present in 5 (28%) of the 18 subjects. None of the subjects in our study had a history of varicella vaccination. The median duration of follow-up was 10 months (range, 5–27 months).

**Table 1:** Demographic profile and systemic evaluation of young patients with herpes zoster ophthalmicus

Case	Age (years)	Sex	Marital status	Occupation	HIV status	AIDS-related complex	Other systemic diseases
1	22	Female	Single	Teacher	Positive	–	Pulmonary tuberculosis
2	49	Male	Married	Shopkeeper	Positive	–	–
3	34	Male	Married	Laborer	Negative	–	–
4	22	Male	Single	Student	Negative	–	–
5	37	Male	Married	Businessman	Positive	–	Syphilis
6	32	Male	Married	Farmer	Positive	+	–
7	27	Male	Married	Government Service	Positive	+	-
8	34	Female	Married	Housewife	Positive	–	Vaginal infection: Candida
9	21	Male	Married	Laborer	Negative	–	–
10	32	Male	Married	Businessman	Negative	–	–
11	29	Male	Single	MBA student	Negative	–	–
12	23	Male	Single	Army Officer	Negative	–	–
13	34	Female	Married	Housewife	Negative	–	–
14	28	Male	Single	IT Professional	Positive	+	Syphilis
15	30	Male	Married	Government Service	Positive	–	Pulmonary tuberculosis
16	34	Male	Single	Trader	Negative	–	–
17	33	Female	Married	Housewife	Negative	–	–
18	24	Female	Married	Housewife	Negative	–	–

HIV: Human immunodeficiency virus, AIDS: Acquired immunodeficiency syndrome, HZO: Herpes zoster ophthalmicus

The clinical features and characteristics of rashes are summarized in Table 2. All subjects presented with skin lesions consistent with zoster involving the ophthalmic division of the trigeminal nerve on the affected side (left side affected in 61% subjects) of the face and head. It was limited to the ophthalmic division in 13 (72%) of 18 subjects. We noted disseminated zoster lesions involving the cervical and thoracic dermatomes in 5 (63%) of the 8 HIV-positive cases and in none of the HIV-negative cases.

**Table 2:** Characteristics of the rash at presentation and chronic sequelae in relation to the immune status of patients with herpes zoster ophthalmicus

Case	HIV status	Duration of rash (h)	Side affected	Dermatomal involvement	Final outcome	PHN (3 months)
1	Positive	24	Left	Multiple (V_1_, C_2–4_, T_6–8_)	UL cicatricial entropion; adherent leukoma	Present
2	Positive	48	Left	Multiple (V_1_, C_6–7_, T_8–10_)	None; adherent leukoma	Present
3	Negative	48	Right	Single (V_1_)	None	Absent
4	Negative	36	Left	Single (V_1_)	Nebular CO	Absent
5	Positive	36	Right	Single (V_1_)	Cicatricial ectropion LL; leukomatous CO	Present
6	Positive	48	Left	Multiple (V_1_, C_2–4_, T_6–8_)	Cicatricial entropion UL; moderate dry eye	Present
7	Positive	24	Right	Single (V_1_)	Cicatricial entropion UL; nebulo-macular CO	Absent
8	Positive	72	Left	Single (V_1_)	Adherent leukoma	Absent
9	Negative	72	Left	Single (V_1_)	None	Absent
10	Negative	24	Right	Single (V_1_)	None	Absent
11	Negative	72	Left	Single (V_1_)	Nebulomacular CO	Absent
12	Negative	36	Right	Single (V_1_)	Nebulomacular CO	Present
13	Negative	48	Left	Single (V_1_)	None	Absent
14	Positive	24	Left	Multiple (V_1_, C_4–6_, T_6–10_)	Cicatricial ectropion LL	Present
15	Positive	72	Left	Multiple (V_1_, C_7_, T_6–9_)	Lid coloboma; focal loss of cilia	Present
16	Negative	36	Left	Single (V_1_)	None	Absent
17	Negative	48	Right	Single (V_1_)	None	Absent
18	Negative	48	Right	Single (V_1_)	Nebulomacular CO	Absent

HIV: Human immunodeficiency virus, PHN: Postherpetic neuralgia, V_1_: Ophthalmic division of trigeminal, C: Cervical dermatome, T: Thoracic dermatome, UL: Upper lid, LL: Lower lid, CO: corneal opacity

Serological tests had confirmed 8 (44%) of the 18 subjects were HIV positive. None of them had previously been diagnosed with HIV infection. Serological testing for HIV was repeated in 4 previously negative subjects after 6–9 months of the first test. The repeat test was negative in the 4 subjects. Serological tests for syphilis (VDRL) were positive in 2 subjects. Chest X-ray findings and tuberculin skin test were confirmatory for tuberculosis in another 2 subjects. Results of other systemic investigations including the complete hemogram with peripheral smear, blood sugar profile, and serological tests for Hepatitis B and C were negative for the entire cohort. Concomitant systemic diseases were present in 5 subjects: tuberculosis (2 subjects); syphilis (2 subjects); and vaginal candidiasis (1 subject); all of whom were serologically positive for HIV. There was no evidence of co-existing systemic disease in the HIV-negative group.

Ocular features of the young subjects are detailed in Table 3. Visual acuity was impaired in all subjects at presentation, the impairment ranging from 20/40 to 20/1200. The majority of the subjects who were HIV positive (88%) had visual acuity of 20/200 or worse at the initial visit and 60% of the immunocompetent subjects with HZO had visual acuity of 20/80 or better at presentation. Conjunctival hyperemia and lid edema ranging from mild to severe was observed in all subjects in the acute phase. Corneal involvement was seen in 15 (89%) of the 18 subjects in the form of dendritic keratitis (28%), punctate keratopathy (28%), stromal keratitis (22%), and peripheral ulcerative keratitis (6%). Corneal sensitivity was abnormal in 78% of cases. Nine subjects (50%) presented with uveal inflammation, which was accompanied with secondary glaucoma in 5 subjects (28%). Posterior segment evaluation revealed cytomegalovirus retinitis in 2 HIV-positive subjects and optic neuritis in 2 HIV-negative subjects. Ocular motility was altered in 2 HIV-negative subjects who presented with fourth nerve palsy.

**Table 3:** Ocular features at presentation and final visual acuity in young adults herpes zoster ophthalmicus

Case	HIV status	Initial BCVA	Lid edema	Corneal findings	Corneal sensation	Uveal inflammation	Posterior segment	Final BCVA
1	Positive	20/200	++	Stromal keratitis, superimposed bacterial keratitis	-	+	WNL	20/400
2	Positive	20/100	++	Dendritic keratitis, superimposed bacterial keratitis	-	++	WNL	20/1200
3	Negative	20/60	+	Punctate keratitis	-	None	WNL	20/30
4	Negative	20/80	++	Dendritic keratitis	-	+	WNL	20/30
5	Positive	20/400	++	Stromal keratitis	-	++	WNL	20/200
6	Positive	20/1200	++	Stromal keratitis	-	++	CMV Retinitis	20/400
7	Positive	20/400	++	None	+	None	CMV Retinitis	20/200
8	Positive	20/200	++	Neuroparalytic keratitis	-	None	WNL	20/400
9	Negative	20/60	++	Exposure keratopathy	-	++	WNL	20/40
10	Negative	20/60	++	Punctate keratitis	+	None	WNL	20/30
11	Negative	20/80	++	Dendritic keratitis	–	++	WNL	20/30
12	Negative	20/200	++	Peripheral ulcerative keratitis, dendritic keratitis	–	++	WNL	20/30
13	Negative	20/200	+	None	–	None	Optic neuritis	20/40
14	Positive	20/800	++	Punctate keratitis	–	None	WNL	20/200
15	Positive	20/400	++	Central stromal keratitis, corneal thinning	–	++++	WNL	20/200
16	Negative	20/400	++	Peripheral ulcerative keratitis with uveal prolapse	–	++	WNL	20/40
17	Negative	20/800	-	None	+	–	Optic neuritis	20/80
18	Negative	20/80	+	Dendritic keratitis	+	–	WNL	20/30

HIV: Human immunodeficiency virus, BCVA: Best corrected visual acuity, WNL: Within normal limits, CMV: Cytomegalovirus

Young adults with no evidence of HIV infection presented with a localized, less severe form of the disease, which were more responsive to therapy. In this series of subjects, manifest corneal and uveal inflammation developed in majority (89%) of the affected eyes, but resolved completely with prompt medical management. None of the immunocompetent subjects developed persistent bullous keratopathy or refractory corneal ulceration or perforation. The final visual acuity in immunocompetent subjects was 20/40 or better in 9 (90%) subjects. Nebulomacular corneal opacities were seen in 5 immunocompetent (50%) subjects. Complete resolution of optic neuritis and fourth nerve palsy was seen at the final visit in the 3 HIV-negative cases.

In the HIV-positive group, the final visual acuity was 20/200 or worse in all subjects. The hypesthetic and dry corneal surface led to secondary bacterial keratitis in 2 of the 8 HIV-positive subjects (25%), contributing to poorer visual outcome in this group. *Staphylococcus aureus* was isolated from the smears collected from the first case and coagulase-negative *Staphylococcus* was implicated in the second case. Chronic sequelae in the form of cicatricial lid deformities, entropion, ectropion, and acquired lid coloboma with focal loss of cilia, eyelid scars were exclusively present in HIV-positive subjects (75%). Postherpetic neuralgia (PHN) was noted in 6 (75%) of the 8 HIV-positive subjects and in 1 (10%) of the 10 HIV-negative subjects.

All the subjects with active herpes zoster ophthalmicus, both HIV negative and HIV positive were prescribed oral acyclovir 800 mg 5 times a day for 2 weeks.

## DISCUSSION

Herpes zoster is uncommon in adults younger than 40 years and the peak incidence occurs in the fifth to the seventh decade of life. The annual incidence of herpes zoster in adults 20–40 years old, is 1.2 per 1000 person years compared with 9.4 per 1000 person-years in adults above 80 years of age in the United States.^6^ Similarly, the incidence of herpes zoster in the European primary care population was the lowest in young adults (1.9/1000 person-years in <44 years old).^7^ The disease spectrum and clinical features in adults younger than 40 years has not been extensively described. Our study highlights the clinical and demographic profile of HZO in this age group and also aims to correlate the clinical manifestations and final outcome with the immune status.

Clinically, many believe that the occurrence of HZO at such a young age is associated with an underlying HIV infection.^8^ The normal spectrum of HZO in immunocompetent individuals has been described by Zaal *et al*.,^9^ where 23 patients were younger than 50 years of age and 50 patients were older than 50 years. In Zaal *et al*.,^9^ prospective study, the immunocompetent young adults (<50 years) formed 31% of the study group, but their unique clinical features and disease spectrum were not highlighted. HZO has also been reported in immunocompetent, healthy children with a favorable outcome.^10^ In our study, 56% of the cases had no clinical evidence of immunosuppression.

On comparing the clinical features and final outcome in these patients in relation to their HIV-seropositive status, several interesting findings emerged. Systemic disease was present in 63% of the patients with HIV-positive status. This is consistent with previous reports of HIV-positive patients who had evidence of concomitant tuberculosis^11^ and syphilis.^4^ None of the immunocompetent patients had evidence of coexisting systemic disease. Patients afflicted with HIV were at an increased risk of developing disseminated cutaneous lesions with a more exuberant cicatrisation response, manifesting as entropion and ectropion of the eyelids. The chronic course of the disease in the HIV-positive patients was noted to be more commonly complicated with secondary bacterial keratitis, poor visual outcome, and PHN.

Young adults who were apparently immunocompetent presented with a localized, less severe form of the disease with better response to medical therapy. The main cause of moderate to severe visual loss at presentation in the young, immunocompetent patients was optic neuritis, but it was reversible with an early course of pulse intravenous steroids and aggressive antiviral therapy. Some degree of reduced corneal sensitivity was present in the majority (70%) of the immunocompetent patients without any associated long-term morbidity.

Upper eyelid scarring has been reported to be a rare manifestation in zoster patients.^12^ The cicatrisation response in the herpetic vesicles was more severe in the HIV-positive group. None of the immunocompetent cases developed similar cicatricial sequelae involving the lids.

Post zoster, the corneal sensation was diminished in 88% of the HIV-positive subjects. This hypesthetic corneal surface, often with an impaired tear secretion, predisposes the cornea to secondary infections. The impaired immunity in cases with HIV further compounds this risk. Similar results of poor visual recovery due to microbial keratitis in HIV-positive patients has been reported.^13^ There was no superimposed bacterial infection in the apparently immunocompetent group despite the diminution of corneal sensations in 70% of these subjects.

The greater severity and prolonged course of HZO, together with superimposed bacterial infection accounted for a poorer visual outcome in the HIV-positive group, and the final visual acuity was poorer than 20/200 in all cases. In contrast, the lesions in the immunocompetent individuals subsided earlier, with a favorable visual outcome (>20/40 in 9 of the 10 subjects).

The incidence of PHN (44%) in our study was quite high as compared with reports in literature.^8^ PHN was reported more commonly in the HIV-positive group in our study, which is similar to some African studies on HIV-related HZO.^4^ The risk of PHN increases with increasing age and severity of the rash^14^ and has been reported to be uncommon in young zoster patients. A study from Iceland^15^ demonstrated variations in risk of developing PHN with different age groups and patients younger than 50 years never developed severe pain at any time. This is in contrast to our study where 75% of the young HIV-afflicted individuals described features suggestive of PHN; 3 months after the rash had subsided.

Our study outlines the spectrum of herpes zoster ophthalmicus in young adults. Prompt diagnosis and early intervention is effective in healthy patients.

However, HZO in young adults may be a harbinger of underlying immunodeficiency with a significant impact on the clinical presentation and final visual outcome.
